# Effect of Peer Counselling by Mother Support Groups on Infant and Young Child Feeding Practices: The Lalitpur Experience

**DOI:** 10.1371/journal.pone.0109181

**Published:** 2014-11-04

**Authors:** Komal P. Kushwaha, Jhuma Sankar, M. Jeeva Sankar, Arun Gupta, J. P. Dadhich, Y. P. Gupta, Girish C. Bhatt, Dilshad A. Ansari, B. Sharma

**Affiliations:** 1 Department of Pediatrics, BRD Medical College, Gorakhpur, India; 2 Department of Pediatrics, AIIMS, New Delhi, India; 3 IBFAN Asia, BP-33, Pitampura, Delhi, India; 4 BPNI, BP-33, Pitampura, Delhi, India; 5 YG Consultants Pvt. Ltd., New Delhi, India; The University of Kansas Medical Center, United States of America

## Abstract

**Objective:**

Our primary objective was to evaluate the effect of peer counselling by mother support groups (MSG's) in improving the infant and young child feeding (IYCF) practices in the community.

**Methods:**

We conducted this repeated-measure before and after study in the Lalitpur district of Uttar Pradesh, India between 2006 and 2011. We assessed the *IYCF practices* before and after creating MSG's within the community. The feeding practices were reassessed at two time points–2 (T1) and 5 years (T2) after the intervention and compared with that of the pre-intervention phase (T0).

**Results:**

The total population covered by the project from the time of its initiation was 105000. A total of 425 (T0), 480 (T1) and 521 (T2) mother infant pairs were selected from this population. There was significant improvement in the following IYCF practices in the community (represented as %; adjOR (95% CI, p) such as *initiation of breast feeding* within 1 hour at both T1 (71% vs. 11%); 19.6 (13.6, 28.2, p = <0.0001)and T2 (62% vs. 11%); 13.3 (9.4, 18.9, p = <0.0001); use of prelacteal feeds at both T1 (67% vs. 15%); 12.6 (CI: 9.0, 17.6, p<0.0001) and T2 (67% vs. 5%); 44.4 (28.8, 68.4, p = <0.0001); *rates of exclusive breast feeding for 6 months* at both T1 (50% vs. 7%); 13.6 (7.6, 25.0, p = <0.0001) and T2 (60% vs. 7%); 20.5 (11.3, 37.2, p = <0.0001); *initiation of complementary feeding* at T1 (85% vs. 54%); 5.6 (3.6, 8.7, p = <0.0001) and T2 (96% vs. 54%); 22.9 (11.8, 44.1, p = <0.0001) and *complementary feeding along with continued breast feeding* at both T1 (36% vs. 4.5%); 6 (1.15, 31.4, p = 0.033) and T2 (42% vs. 4.5%); 8.06 (1.96, 49.1, p = 0.005) as compared to pre-intervention period (T0) after adjusting for important social and demographic variables.

**Conclusions:**

Peer counseling by MSG's improved the IYCF practices in the district and could be sustained.

## Introduction

India has the highest number of under-five child deaths in the world. The current under- five mortality rate (U5MR) of India is 52 per 1000 live-births [1]. Malnutrition may be the underlying cause in up to 50 per cent of these under-five deaths [2]. Exclusive breastfeeding during the first six months and continued breastfeeding for two years and beyond along with introduction of appropriate complementary foods at six months could contribute significantly in reducing childhood malnutrition and improving child survival [3]. Breastfeeding is an evidence based intervention that has been found to significantly reduce the neonatal and thereby the child deaths. It has been estimated that, breast feeding alone may reduce the child mortality by 13% [4].

On the other hand, complementary feeding to support it has been shown to reduce stunting significantly [5]. To put it simply, promotion of optimal feeding practices improves the breast feeding rates and thereby the child mortality. It also ensures better complementary feeding which indirectly improves the under nutrition and thereby the child mortality. Therefore, the key to child survival in a country like ours with such high under five and infant mortality rates is to ensure Optimal Infant and Young Child Feeding (IYCF) practices.

Promotion of optimal IYCF practices in the community could be done by various kinds of supportive relationships such as social/lay support or professional support. While professional support may reach out to limited population attending the health care facilities for health related issue, social/lay support will reach out to the entire community. Two main types of social support networks exist [6], [7]. One, the embedded social networks- comprising of family members/friends, church members, co-workers, neighbors etc. and the second, the created social networks which are most often used for promotion of various type of preventive measures in the community[7]. Thus the type of support may range from pure lay person support to only professional support. The different types of created social networks include self-help groups (no professional involvement), support groups (with the help of professionals) and para-professionals (extensive training and professional involvement). The aim of the support groups is to provide **peer support**. Peer support is defined as ‘the provision of emotional, appraisal and informational assistance by a created social network member who possesses experiential knowledge of a specific behavior or stressor and similar characteristics as the target population’ [7]. Peer supporters could be either voluntary or in receipt of basic remuneration or money for expenses.

The WHO and UNICEF recognize peer support and counselling as an important component of policies and programs to support breastfeeding [8], [9], [10]. The Baby Friendly Hospital initiative (BFHI) has been endorsed by both of these organizations for this very purpose [8], [9]. The 10^th^ step of the BFHI which recommends fostering the establishment of breast feeding support groups/peer support groups and referring mothers to them on discharge- is increasingly being recognized as the most crucial link between initiation and continuation of breast feeding in the community. Initiation of breastfeeding can be done by training the professionals in the clinics and hospitals. However, continuation of breastfeeding will depend on factors such as feeding difficulties faced by the mothers after discharge, the amount of support and encouragement the mothers receive to continue exclusive breastfeeding at home and other queries related to feeding that the mothers would have. All of this is possible only with the help of peer support systems within the community. However, this is not an easy task as it requires sustained, systematic and long term effort.

Few studies and recent meta-analyses on effect of peer support have demonstrated beneficial effects of providing peer support on exclusive and any breast feeding rates [11]–[21]. However, it is not clear from the large body of evidence, the aspects of support that are most important [12]. Different forms of support in different contexts will be differentially effective. What works in a particular setting may not work for another! Therefore, it is suggested that the type of support should be indigenous and developed through formative research in the target community [12]. In this context, the concept of Mother Support Groups (MSGs) has not been fully explored. Peer support groups operated by trained mothers, mother support groups operated by women in collaboration with health/nutrition professionals, and mother-to-mother support groups' operated by mothers are three different forms of mother support. Published data on effect of “breast feeding/Mother Support Groups” on IYCF practices are encouraging but a large community intervention has not been implemented till date [11]–[21]. There is only one previous study on peer support from India by Bhandari et al. [20]. The training strategy used was based on Integrated Management of Neonatal and Childhood Illnesses [IMNCI} and trained child health and nutrition workers were used for the purpose of providing peer support. In the other study from Bangladesh by Haider et al. [21], trained local mothers were used. A combination of local mothers in collaboration with health/nutrition workers has not been previously explored. Such a form of group support would ensure quality control as well as ensure sustainability. Therefore, we undertook this study with an aim to assess the feasibility, efficacy and sustainability of this kind of support on the important IYCF practices in the community.

## Materials and Methods

### Design and setting

This was a quasi-experimental before and after study conducted over a period of 5 years from December 2006 and 2011 by the department of pediatrics, BRD Medical College, Gorakhpur in collaboration with UNICEF Office of Uttar Pradesh. The study was approved by the Institutional Ethics Committee of BRD Medical College, Gorakhpur and we obtained written informed consent of all the mothers who participated in the study. The study was undertaken in the 6 blocks of district Lalitpur, one of the most backward districts of Uttar Pradesh with a population of over one million and more than 30,000 annual births. It is one of the most underdeveloped districts of Uttar Pradesh with high rates of under-nutrition, and poor child health indicators [22].

### Purpose and objectives

The primary objective of our study was to evaluate the effect of counselling by MSGs, at 2 pre-decided time points (after 2 year and 5 years of intervention), on IYCF practices such as 1) initiation of breast feeding within 1 hour of birth 2) use of pre-lacteal feeds 3) exclusive breast feeding upto 6 months 4) timely introduction of complementary feeding and 5) complementary feeding along with continued breast feeding up to 2 years of age [3]. Our secondary objectives were to evaluate the effect of counselling by MSGs on 1) proportion of mothers needing support from family members, doctors and others for feeding difficulties 2) mothers' confidence in feeding and 3) effect on long –term outcome such as feasibility of scale up/extension of such projects to other districts of UP as well as other states in India.

### Study population

The study population comprised of mother infant pairs. These included mothers who had delivered a child within 0–3, 3–6, 6–12 and 12–24 months.

### Study intervention/strategy

**Figure 1:**
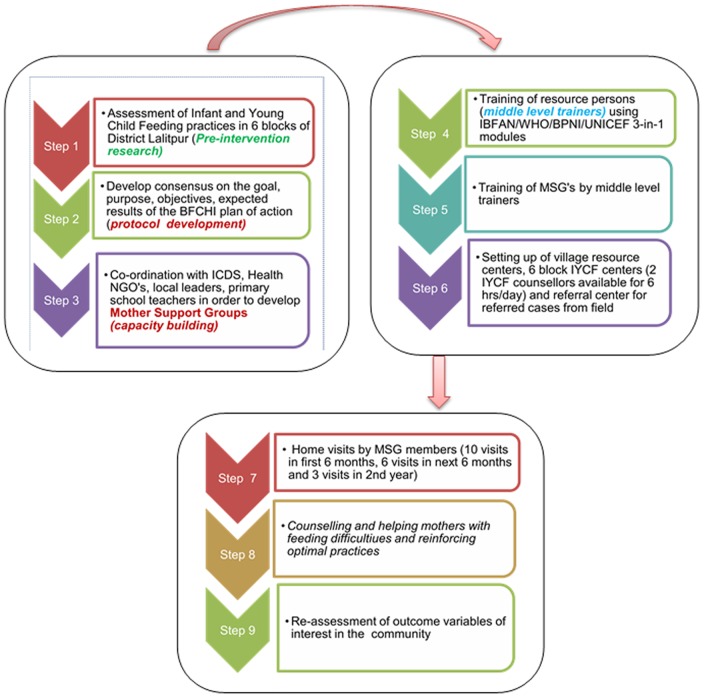
Flow diagram of project activities. MSG, Mother Support Groups; ICDS, Integrated Child Development Services; NGO's, Non-Government officials; BFCHI, Baby Friendly Community Health Initiative; IBFAN, International Baby Food Action Network; WHO, World Health Organization; BPNI, Breast feeding Promotion Network of India; UNICEF, United Nations Children's Fund; IYCF, Infant Young Child Feeding.

The intervention comprised of counselling and providing support to these mothers by the MSGs. Both facility based and community based strategy were used. A flow diagram of the project activities is briefly depicted in Figure 1.

We initially conducted a rapid assessment of IYCF practices by door to door survey in all the six blocks in November 2006. Following this, the study protocol was developed and the goals, objectives and expected results were finalized. The next step was to identify potential candidates for the MSG's. This was done with the coordination of Integrated Child Development Schemes (ICDS), Non- Government Officials (NGOs), local leaders and primary school teachers. This was followed by deploying and training the resource persons for the project. In the period between January and December 2007, a total of 48 ‘facility/block level trainers' were selected from 6 blocks (8 per block) and trained over a 7 day period using the International Baby Food Action Network (IBFAN)/WHO/UNICEF/Breast Feeding Promotion Network of India (BPNI) 3-in-1 training modules (an integrated course on breastfeeding, complimentary feeding and infant feeding and HIV) [23]. These facility level trainers in turn trained Anganwadi workers, ASHAs (ICDS and Health frontline functionaries) and *Dais* (traditional birth attendants) in the district in a three day workshop. These trainings were monitored by BPNI, UNICEF, and project implementation officials. A total of 1847 women thus trained were responsible for setting up ‘Mothers Support Groups' in 600 ICDS villages of the district in a period of 12 months. The MSGs comprised of 3–4 members of a group comprising of a traditional birth attendant, experienced mother, and community health/nutrition worker (Anganwadi worker or AWW). The MSG's were trained in basic communication skills and in infant and young child feeding. They were under direct supervision of facility level trainers. The MSG group members paid home visits to the mother- infant pairs- 10 visits in first 6 months, 6 in next 6 months and 3 in 2^nd^ year. The primary work of the MSGs was to promote and support optimal infant and young child feeding. At least one member from the mother support group was available 24 hours a day for counselling and providing practical help to mothers in need. They arranged meetings with pregnant and lactating mothers once in a week (Saturday) at the center. During these meetings, they provided education on infant and young child feeding and helped and supported mothers with feeding difficulties. In cases of failure, the mothers would be referred to block level counselling centers or district level centers. Each member of MSG received a token amount of Rs. 50 per month (Rs. 150 for 3 workers in each village per month) for their services.

In addition to the setting up of village resource centers, 6 block IYCF resource centers were established in the block hospital premises, where 2 IYCF counselors were available for counselling support for a period of 6 hours each day. One referral center was established in the District Female Hospital for cases referred from the field. The plan of action had an inbuilt capacity building, monitoring, and institutional ethics committee input. Regular review meetings chaired by the District Magistrate and all line department's heads present were held at periodic intervals. To bring together frontline functionaries and all involved in the implementation of the BFCHI project, two large gatherings (mela's) were organized with stalls depicting the actual field based actions. Deserving MSG members were appreciated and honored for morale boosting purposes.

Apart from this, the other activities of the project included imparting advice on kangaroo mother care, helping mothers in preparing good complementary food for babies, feeding skills for complementary feeding, weekly health and nutrition day (Saturday), village health and nutrition day (once a month), immunization and use of MCP cards and recording of births and deaths. A detailed report on the methodology and the project activities in the district can be accessed at http://www.bpni.org/BFHI/Reaching-the-under-2S-Universalising-Delivery-of-Nutrition-Interventions-in-Lalitpur-UP.pdf [24].

### Data collection and study period

A base line survey (T0) was done in the 6 blocks of the district in November 2006 and 421 mothers were interviewed in the pre-intervention period. Data collection in the baseline survey was started in the 2nd week of November, 2006 and was completed by the last week of November, 2006. In the post-intervention periods at T1 (Jan 2008) and T2 (Dec 2011), 480 and 597 mother infant pairs respectively were selected to see the impact of the intervention. Local male and female field investigators and volunteers of BFCHI project, Lalitpur were recruited and trained for collecting information from mothers in selected villages and village level counselors. Data was collected during all the three periods by door to door survey using the same set of questionnaire. The persons collecting data were trained for a period of 1 month prior to data collection each time by the principal investigator and the queries resolved. All the field investigators/supervisor for this study were thoroughly oriented about the questionnaire/schedule used for quantitative data collection. Following this, for 1 month, the data collected (twice in each case) by the project staff were checked for accuracy and any inconsistencies were resolved. The master chart as well as individual proformas' of the data collected at the three time points can be accessed at the project office (Department of Pediatrics, Gorakhpur Medical College, UP, India).

### Sample size and sampling design

The sample size was estimated based on the expected prevalence of exclusive breastfeeding rates in the district at each time point. In the pre-intervention stage with assumed prevalence of EBF of 40% and absolute precision of 5% and a design effect of 1.2 (for clustering), the sample size calculated was 427. The corresponding sample sizes for the 1^st^ and 2^nd^ post intervention stages were 447 and 440 respectively, assuming the same design effect and expected prevalence of EBF to be 50% and 55% in the two time periods.

At the first stage, panchayats were selected from each block using a PPS sampling technique. Thus, in all the 6 blocks were selected for collecting information from mothers. At the second stage, mothers who have delivered a child within 0–3 months, 3–6, 6–12 and 12–24 months were selected from the sample villages. Thus, a total sample of size 421 (base line), 480 (second phase) and 597 (third phase) mothers with children between zero to twenty four months were selected from the 6 blocks and interviewed to assess the initiation of breastfeeding and complementary feeding practices in the area. In case sample was not achieved in any particular village, adjoining village was considered to achieve the required sample.

### Statistical analysis

Data was entered into MS Access and analyzed using Stata 11. Categorical data are presented as number (%) while continuous variables are presented as mean (SD), if normally distributed and median (interquartile range), if skewed. Statistical analysis was performed using Student's t-test/Wilcoxon rank sum test and Chi-square test for continuous and categorical variables respectively. We calculated the relative risk (RR)/mean difference with 95% CI for all the outcomes. P-value of <0.05 was considered significant.

For determining effect of the intervention on ‘each of the IYCF practices' at both time points after adjusting for other socioeconomic and demographic variables prevalent at the time, we performed multivariable logistic regression with backward stepwise elimination variable selection method using “presence of each IYCF practice” as the dependent variable and all others as independent variables. The results of the multivariable analysis are reported as adjusted odds ratios (adjOR) with 95% CI. If the IYCF practice was followed at the time, we took it as ‘yes’ or else as ‘no’.

## Results

The total population covered by the project was 105000. The project reach was universal with more than 84–90% mothers having received advice on various feeding practices by one or more of the MSG members.

The baseline characteristics of the study population during the three time periods are described in Table 1. There were no significant differences between the three time periods with regard to demographic and social factors such as age, educational level, occupation or caste. Majority of the women were homemakers in their 20′s and were illiterate. One quarter of the women actively worked in agricultural fields during the day.

**Table 1:** Demographic and socio-economic profile of mothers during both time periods.

Variables	Baseline survey T0; (N = 421)	Post-intervention Survey 1; T1(N = 480)	Post-intervention survey 2; T2 (N = 597)
**Age in years**			
≤19	25 (6)	19 (6)	25 (4)
20–29	370 (88)	432 (90)	535 (90)
30–39	26 (6.2)	19 (4)	37 (6)
**Educational level**			
Illiterate	215 (51)	235 (49)	296 (50)
Primary school	96 (23)	120 (25)	158 (26)
Middle school	67 (16)	86 (18)	112 (19)
High school	29.5 (7)	38 (8)	31 (5)
Intermediate	13 (3)	-	-
Graduate and above	4 (1)	-	-
**Occupation**			
Housewife	265 (63)	331 (69)	401 (67)
Agriculture	122 (29)	100 (21)	149 (25)
Govt. Service	4 (1)	10 (2)	15 (3)
Daily wages/Laborer	25 (6)	38 (8)	28 (5)
Unskilled Worker	1 (0.02)	-	-
Skilled worker	1 (0.02)	1 (0.02)	4 (0.07)
**Occupation of father**			
Agriculture	197 (47)	213 (44)	311(52)
Self-employed	41 (10)	62 (13)	82 (14)
Skilled worker	43 (10)	78 (3)	51 (8)
Unskilled worker	104 (25)	103 (22)	95 (16)
Unemployed	36 (9)	24 (5)	58 (10)
**Religion**			
Hindu	419 (99.5)	469 (98)	561 (94)
Muslim	2 (0.05)	11 (2)	36 (6)
**Caste**			
SC	105 (25)	168 (35)	227 (38)
ST	42 (10)	34 (7)	30 (5)
OBC	231 (55)	219 (46)	301 (50)
Others/Higher Caste	42 (10)	59 (12.3)	39 (6.5)
**Age group of infants**			
0–2.9 months	110 (26)	123 (26)	185 (31)
3–5.9 months	48 (11)	71 (15)	133 (22)
6–11.9 months	219 (52)	233 (49)	207 (35)
12–24 months	44 (10)	53 (11)	72 (12)

Data presented as number (percent) unless otherwise indicated

T0, baseline; T1–2 year after intervention; T2–5 years after intervention; SC, Scheduled Caste; ST, Scheduled Tribe;

OBC, Other Backward Classes.

### Primary outcomes

T0 vs. T1: The intervention had significant effect on theinfant and young child feeding (IYCF) practices evaluated at the first re-assessment (T1) (Table 2). All the practices evaluated improved after the intervention (p<0.001 for all).

**Table 2:** Infant and young child feeding practices in the community before and after the intervention.

Feeding parameter	Pre- intervention (T0) N = 421	Post-intervention (T1) N = 480	T0 vs. T1 Unadjusted OR (95% CI); P	Post- intervention (T2) N = 597	T0 vs. T2 Unadjusted OR (95% CI); P
Initiation of breastfeeding kwithin one hour of birth	46/421 (11%)	340/480 (71%)	19.8 (13.7, 28.5); p<0.0001	370/597 (62%)	13.3 (0.4, 18.8); p<0.0001
No use of pre-lacteal feeds	139/421 (33%)	408 (85%)	11.5 (8.3, 15.8); p<0.0001	567/597 (95%)	38.3 (25.2, 58.3); p<0.0001
Exclusive breastfeeding for 6 months	15/219 (7%)	117/233 (50%)	13.7 (7.6, 24.6); p<0.0001	124/207 (60%)	20 (10.7, 39); p<0.0001
Initiation of complementary feeding (6–8 months)	143/263(54%)	243/286 (85%)	4.7 (3.16, 7.1); p<0.0001	268/279 (96%)	20.4 (10.7, 39.2); p<0.0001
Complementary foods along with continued breastfeeding upto 2 year	2/44 (4.5%)	19/53 (36%)	11.7 (2.6, 54); p = 0.002	30/72 (42%)	15 (3.36, 66.8); p<0.0001

T0, baseline; T1–2 year after intervention; T2–5 years after intervention.

T0 vs. T2: Similar effect was seen at the 2^nd^ re-assessment (T2) with all the IYCF practices showing significant improvement over the baseline data (p<0.001 for all; Table 2).

The improvement in the IYCF practices remained significant even after adjusting for socioeconomic and demographic variables such as mothers age, education level of mother, religion, caste, and fathers occupation - such as *initiation of breast feeding* within 1 hour at both T1 (71% vs. 11%); adjOR: 19.6 (95% CI: 13.6, 28.2, p = <0.0001)and T2 (62% vs. 11%); adjOR: 13.3 (95% CI: 9.4, 18.9, p = <0.0001); no use of prelacteal feeds at both T1 (67% vs. 15%); adjOR: 12.6 (95% CI: 9.0, 17.6, p<0.0001) and T2 (67% vs. 5%); adjOR: 44.4 (95%CI: 28.8, 68.4, p = <0.0001); *rates of exclusive breast feeding for 6 months* at both T1 (50% vs. 7%); adjOR: 13.6 (95% CI: 7.6, 25.0, p = <0.0001) and T2 (60% vs. 7%); adjOR: 20.5; (95% CI: 11.3, 37.2, p = <0.0001); *initiation of complementary feeding* (6–8 months) at T1 (85% vs. 54%); adjOR: 5.6 (95% CI: 3.6, 8.7, p = <0.0001) and T2 (96% vs. 54%); adjOR: 22.9 (95% CI: 11.8, 44.1, p = <0.0001) and *complementary feeding along with continued breast feeding up to 2 years of age* at both T1 (36% vs. 4.5%); adjOR: 6 (95% CI: 1.15, 31.4, p = 0.033) and T2 (42% vs. 4.5%); adjOR: 8.06 (95% CI: 1.96, 49.1, p = 0.005) as compared to pre-intervention period (T0) (Table 3).

**Table 3:** Effect on any IYCF practices: multivariate analysis*.

IYCF practices	Baseline vs. T1	Baseline vs. T2
	*Adjusted OR (95% CI)* * *; P value*	
**Initiation of BF**	19.6 (13.6, 28.2); p = <0.0001	13.3 (9.3, 18.8); p = <0.0001
**No use of pre-lacteal feeds**	12.7 (9.0, 17.7); p = <0.0001	44.4 (28.8, 68.4); p = <0.0001
**Exclusive BF**	13.6 (7.6, 24.6); p = <0.0001	20.5 (11.3, 37.2); p = <0.0001
**Initiation of CF**	5.6 (3.6, 8.7); p = <0.0001	22.8 (11.8, 44.1); p = <0.0001
**Continued BF till 2 yr**	6.02 (1.15, 31.4); p = 0.033	9.8 (1.96, 49.1); p = 0.005

*Adjusted for mother's age, mother's education, religion, caste, father's occupation.

### Secondary outcomes

The secondary outcomes such as mother's confidence, responsive feeding and help seeking behavior of mothers for feeding also improved after the intervention, with the difference between the baseline data and data collected at both time points, *i.e*. T1 and T2 being statistically significant for all outcomes (Table 4). After the intervention, majority of mothers (42–47%) were seeking help from trained workers rather than family members (8–20%). The confidence of mothers about their breast milk being sufficient and responsive feeding also improved after the intervention (Table 4).

**Table 4:** Secondary outcomes of the study population.

Parameter	Pre- intervention (T0) N = 421	Post-intervention (T1) N = 480	T0 vs. T1 Unadjusted OR (95% CI); p	Post- intervention (T2) N = 597	T0 vs. T2 RR (95% CI); p
Not seeking help from family members	264 (62%)	382 (80%)	2.31 (1.72, 3.12); p<0.0001	549 (91%)	6.8 (4.8, 9.7); p<0.0001
Help by Trained worker/ANM/Dai/Doctor	35 (8%)	200 (42%)	7.9 (5.3, 11.6); p<0.0001	280 (47%)	9.74 (6.7, 14.3); p<0.0001
Help by doctor	21 (5%)	14 (3%)	0.57 (0.3, 1.1); 0.05	58 (10%)	2.0 (1.2, 3.4); p = 0.002
Mother's belief about her milk supply as sufficient (confidence)	63 (15%)	192 (40%)	3.8 (2.7, 5.2); p<0.0001	327 (55%)	6.9 (5.0, 9.4); p<0.0001
Self -stopping of sucking of child during feeding (responsive feeding)	83 (20%)	315 (65%)	7.8 (5.7, 10.6); p<0.0001	330 (55%)	5.0 (3.8, 6.7); p<0.0001

T0, baseline; T1–2 year after intervention; T2–5 years after intervention.

### Feasibility for scale-up/extension to other districts of the state

The project during it course was able to develop 1286 MSG's which involved 3858 local women (ASHA's-767; AWW's-1124, Helpers-287; trained mothers –1680). We also trained 18 block level counselors; conducted 655721 counselling sessions with>100000 of these being facility based. The project reach was universal with 84–90% of mothers reporting having received advice on various infant feeding practices by AWW's (63–67%), ASHA's (16–21%) and Dai's (5–16%). Currently all the above activities are firmly in place and have been integrated into the health system of District Lalitpur and other adjoining districts such as Jhansi.

## Discussion

Our study findings (one of the largest community based study) have demonstrated that the IYCF practices evaluated in the study can be significantly improved in the community by using local resources, by creating MSGs from within the community and the activities may be sustained by integrating the activities into the health policies of the district/state. Our study has also demonstrated that a community intervention on improving the evaluated IYCF practices can be implemented successfully in a population of over 1 million and may even be sustained for a short term such as 5 years. The improvement in IYCF practices remained significant even after adjusting for important social and demographic variables during both post-intervention periods (T1 and T2) such as, mother's age and education, father's occupation, caste and, religion. The possible reason for no visible effect of these factors could be that the proportion of Individual subclasses wasfairly constant in the district with minor variations not significant enough to affect the outcomes. At the beginning, most mothers irrespective of class, caste or religion had poor knowledge of IYCF practices and were not seeking appropriate help for their feeding problems. Through the intervention and counselling by the MSG's, it improved over time and was sustained. Our findings are reflective of a recently published survey on IYCF practices in the country [25]. In this survey on analysis of socioeconomic factors that contribute to poor feeding practices, the author observed a strong correlation, only between nutritionaladvice on infant feeding practices by health care workers across all age groups. The mother's wealth status was found to have only a small effect on feeding practices.

Two major strategies were adopted for the project including a community based and a facility-based strategy. The implementation mechanisms included providing infant feeding counseling at village, block and district level; administrative meetings of stakeholders at sector and district level; project review meetings; linkages with ICDS, health and village volunteers and strengthening of immunization program.

Creating a support group with appropriate training and skills in the community improved the help seeking behavior of lactating women in the district. This was clear from the study results which showed that before the intervention, most of the lactating mothers were turning to the family members for the help, who were armed with only their traditional wisdom which many a times does not provide necessary confidence building counseling. Whereas, after the intervention, a significant number of lactating women were seeking help from the trained personnel and relying less on the family members.

Another important factor that improved with our intervention was the mothers' confidence. The intervention led to significant improvement in the number of mothers' believing in the adequacy of their milk supply, good number of them reporting that the child stops sucking by her/himself during the suckling.

Our study findings are supported by similar previous facility based and home/community based peer counsellingstudies [14]–[21]. In the first reported home/community based randomized trial of breastfeeding promotion, 130 pregnant mothers were randomized to receive either one of the two types of counselling frequencies such as six visits and 3 visits or no visits (control group). Home visits were made during pregnancy and early post-partum by peer counselors recruited from the same community (similar to what we did in our study) and trained by a special league meant for this purpose. At 3 months post- partum, exclusive breastfeeding had improved significantly as compared to the control group. Duration of breastfeeding was significantly (p = 0.02) longer in intervention groups than in controls [19]. In comparison to this study, the counselling sessions in our study were mainly targeted at lactating mothers with home visits by MSG members at birth, followed by 9 visits thereafter in first 6 months and 9 visits up to 2 years of age.

In a trial on facility based counselling for promotion of breastfeeding, popularly known as the promotion of breastfeeding intervention trial or the PROBIT trial, the authors hypothesized that promotion of breastfeeding would improve exclusive breastfeeding rates and reduce the incidence of gastrointestinal and respiratory infection among infants. In this trial, a total of 17046 mother-infant pairs were randomly assigned to receive an experimental intervention based on the BFHI model of the WHO/UNICEF or a control intervention of continuing usual infant feeding practices. The counselling was provided by the health care workers of maternity hospitals and polyclinics of Belarus. The results favored the intervention group with infants from intervention sites significantly more likely than control group infants to be breastfed to any degree at 12 months [17]. Although this trial was also based on the BFHI program, the intervention was facility based in this study and not community based as was in our study.

In a study reported form our neighboring country (Dhaka, Bangladesh) [21], the authors trained local mothers over 40 hrs based on the WHO/UNICEF breastfeeding counselling-training course [26]. The intervention involved 15 counselling visits to mothers' homes by these mothers until infants reached five months, starting with two visits in the last trimester of pregnancy, and three in the first two weeks of life. The prevalence of exclusive breastfeeding at five months was 70% for the intervention group, almost similar to what we found in our study after 1 year of intervention.

In the two previous RCTs from India and its neighboring country Bangladesh (see introduction), the authors found similar rates of improvement in breastfeeding practices in the community in the intervention groups as compared to the control groups with the use of peer counselling. However, the strategies used in these studies as described in the introduction, were different from ours.

These studies, including ours highlight the importance of good practical skill training, counselling, the presence of local women in the support group, skilled supervision, a supportive health system and administration, the presence of referral centers and timely referral, frequent post-natal contacts (eight to nine), regular orientation, refresher courses, and incentives for the work. Several systematic reviews and meta-analyses have also been carried out on this subject [11], [12], [27]. For example, in a recent meta-analyses on effect of peer support on duration of exclusive breastfeeding (EBF) in low and middle-income countries (LMICs) the authors observed that peer support significantly decreased the risk of discontinuing EBF as compared to control (RR: 0.71; 95% CI: 0.61–0.82; I2 = 92%) [11]. However, the effect appeared to be reduced in formula feeding cultures. The analyses included eleven randomized controlled trials conducted at 13 study sites [11]. In another meta-analyses published recently by Renfrew and colleagues [12], 67 research reports from 21 countries involving 56,451 mothers receiving breastfeeding support for variable periods were examined. The authors found a positive impact on exclusive breastfeeding (in particular) and any breastfeeding rates in all of these studies. A combination of community-level mother support (lay support) and professional support is more effective than professional support alone, the authors concluded.

In another systemic review in which the search was limited to articles published in Finnish, Swedish, and English in the 2000–2006 period examined breastfeeding support interventions [27]. Interventions that covered the period from pregnancy through to the postnatal period were more effective than interventions of short duration. The effective interventions in pregnancy were interactive ones, such as conversation. Home visits, telephone support, and breastfeeding centers combined with peer support were found to be the most effective. In our study also we had used similar methodology of counselling mother's right through the antenatal period till their post natal period and had used all of the methods mentioned above.

Thus there is unequivocal evidence on the importance of peer group/community based counselling in reducing infant/child mortality by improving IYCF practices in the community. The time is not far away when community based counselling would be integrated into facility based management and into health programs and policies of the state and that would make a huge difference to the current IYCF practices and child health indicators across the world.

The strengths of our study are –it is the largest community based study (project covering a population of >1 million) till date on peer counselling and we used local resources form the community and did not use a specialized team for the study purpose. The project was undertaken for evaluating the feasibility of implementation on such a large scale and its sustainability so that it could be used as a model for the rest of the state and the country. The project reach was universal with more than 80% of mothers form the district counseled on more than one occasion. The project has also been able to create over a 1000 MSG's and 6 resource centers which have been put firmly in place and have been integrated into the state health system. In addition, the project has demonstrated real convergence at village level and heightened motivation of workers to prevent malnutrition and morbidity in infants and young children.

The major limitations of the study are it is not a randomized trial and therefore the causality cannot be determined as we did not have a control group. Selection bias and interviewers bias is unlikely as the interviewers were trained to ask specific questions for each practice parameter and not any leading questions. The other limitations were -we evaluated only the four important IYCF parameters and not all as they were objective and easily recalled, and, the project was funded and it may not possible for all communities to follow suit. Whether the project activities would be sustained in the long run-say after 10 years is also yet to be seen.

## Conclusions

The present study demonstrated a significant improvement in the IYCF practices evaluated when pregnant and lactating mothers were supported with skilled counselling. The project interventions have been effective in increasing the initiation of breastfeeding within one hour of birth of baby, exclusive breastfeeding for 6 months, and appropriate start of complementary feeding. The fact that such an intervention could be implemented in a whole district with a population of over a million, using local resource persons indicates that it could be scaled up in other parts of the country also.

## References

[1] istrar General of India (2011) Sample Registration System (SRS) Statistical Report 2011. New Delhi 2013.

[2] CEF (2007) Progress for children report -A statistical review December 2007. http://www.unicef.org/india/media_3766.htm (accessed July 18, 2013)

[3] /UNICEF (2003) Global strategy for infant and young child feeding. Switzerland: WHO. Retrieved from http://www.who.int/nutrition/publications/infantfeeding/9241562218/en/index.htmlDsdsda

[4] Darmstadt GL, Bhutta ZA, Cousens S, Adam T, Walker N, et al (2005) Lancet Neonatal Survival Steering Team. Evidence-based, cost-effective interventions: how many newborn babies can we save? Lancet 365: 977–88. 15767001 10.1016/S0140-6736(05)71088-6

[5] Bhutta ZA, Ahmed T, Black RE, Cousens S, Dewey K, et al. (2008) Maternal and Child Undernutrition Study Group. What works? Interventions for maternal and child undernutrition and survival Lancet 371:417–40Review. 10.1016/S0140-6736(07)61693-6 18206226

[6] Peer support Groups for parents: A literature review. Available at http://www.first5la.org/files/08226_2.3PSG%20Exploratory%20Study%20-%20Lit%20Review%20FINAL_08312012.pdf accessed on 24^th^ Jan, 2014.

[7] Dennis CL (2003) Peer support within a health care context: a concept analysis. Int J Nurs Stud 40: 321–32. Review. 10.1016/s0020-7489(02)00092-5 12605954

[8] WHO/UNICEF (1991) Baby-Friendly Hospital Initiative. Retrieved from http://www.who.int/nutrition/topics/bfhi/en/index.html

[9] WHO/UNICEF (2009) Baby-Friendly Hospital Initiative - revised, updated, and expanded for integral care. Retrieved from http://whqlibdoc.who.int/publications/2009/9789241594967_eng.pdf 23926623

[10] Community-based strategies for breastfeeding promotion and support in developing countries. Available at http://whqlibdoc.who.int/publications/2003/9241591218.pdf?ua=1 accessed on 24^th^ Jan, 2014.

[11] Sudfeld CR, Fawzi WW, Lahariya C (2012) Peer support and exclusive breastfeeding duration in low and middle-income countries: a systematic review and meta-analysis. PLoS One 7: e45143 10.1371/journal.pone.0045143Epub2012Sep18.Review 23028810 PMC3445598

[12] Renfrew MJ, McCormick FM, Wade A, Quinn B, Dowswell T (2012) Support for healthy breastfeeding mothers with healthy term babies. Cochrane Database Syst Rev 16 5: CD001141 10.1002/14651858.CD001141.pub4Review PMC3966266 22592675

[13] Bland RM, Little KE, Coovadia HM, Coutsoudis A, Rollins NC, et al (2008) Intervention to promote exclusive breast-feeding for the first 6 months of life in a high HIV prevalence area. AIDS 22: 883–91 10.1097/QAD.0b013e3282f768de 18427207

[14] Agrasada GV, Gustafsson J, Kylberg E, Ewald U (2005) Postnatal peer counselling on exclusive breastfeeding of low-birthweight infants: a randomized, controlled trial. Acta Paediatr 94: 1109–15. 16188857 10.1111/j.1651-2227.2005.tb02053.x

[15] Quinn VJ, Guyon AB, Schubert JW, Stone-Jiménez M, Hainsworth MD, et al (2005) Improving breastfeeding practices on a broad scale at the community level: success stories from Africa and Latin America. J Hum Lact 21: 345–54. 16113023 10.1177/0890334405278383

[16] Nor B, Zembe Y, Daniels K, Doherty T, Jackson D, et al (2009) PROMISE-EBF Study Group. "Peer but not peer": considering the context of infant feeding peer counselling in a high HIV prevalence area (2009) J Hum Lact. 25: 427–34 10.1177/0890334409341050Epub2009Jul21 19622755

[17] Kramer MS, Chalmers B, Hodnett ED, Sevkovskaya Z, Dzikovich I, et al (2001) PROBIT Study Group (Promotion of Breastfeeding Intervention Trial) Promotion of Breastfeeding Intervention Trial (PROBIT): a randomized trial in the Republic of Belarus. JAMA 285: 413–20. 11242425 10.1001/jama.285.4.413

[18] Aidam BA, Perez-Escamilla R, Lartey A (2005) Lactation counselling increases exclusive breast-feeding rates in Ghana. J Nutr 135: 1691–5. 15987851 10.1093/jn/135.7.1691

[19] Morrow AL, Guerrero ML, Shults J, Calva JJ, Lutter C, et al (1999) Efficacy of home-based peer counselling to promote exclusive breastfeeding: a randomised controlled trial. Lancet 10 353: 1226–31. 10.1016/S0140-6736(98)08037-4 10217083

[20] Bhandari N, Bahl R, Mazumdar S, Martines J, Black RE, et al (2003) Infant Feeding Study Group Effect of community-based promotion of exclusive breastfeeding on diarrhoeal illness and growth: a cluster randomised controlled trial. Lancet 361: 1418–23. 12727395 10.1016/S0140-6736(03)13134-0

[21] Haider R, Ashworth A, Kabir I, Huttly SR (2000) Effect of community-based peer counsellors on exclusive breastfeeding practices in Dhaka, Bangladesh: a randomized controlled trial. Lancet 356: 1643–7. 11089824 10.1016/s0140-6736(00)03159-7

[22] District Level Household and Facility Survey under Reproductive and Child Health Project (DLHS-3)Available at http://www.scribd.com/doc/59581835/37-Revised-Factsheet-Lalitpur-UP accessed on 24^th^ Jan, 2014.

[23] BPNI/IBFAN (2009) The “3 in 1”training program: Capacity building initiative for building health workers' skills in infant and young child feeding counselling training course (BPNI/IBFAN, 2009) Retrieved from http://www.bpni.org/Training/3-in-1-TP-BPNI.pdf

[24] Kushwaha KP (Ed.) (2010) Reaching the under 2s: Universalising the delivery of nutrition. Intervention in District Lalitpur, Uttar Pradesh. Gorakhpur, India: Department of Paediatris, BRD Medical College.

[25] Malhotra N (2013) Inadequate feeding of infant and young children in India: lack of nutritional information or food affordability? Public Health Nutr. 2013 16: 1723–31. 10.1017/S1368980012004065 PMC10271245 22939461

[26] WHO. Breastfeeding counselling: a training course. Geneva: WHO.Retrieved from http://www.who.int/maternal_child_adolescent/documents/who_cdr_93_3/en/index.html.

[27] Hannula L, Kaunonen M, Tarkka MT (2008) A systematic review of professional support interventions for breastfeeding. J Clin Nurs 17: 1132–43 10.1111/j.1365-2702.2007.02239.xReview 18416790

